# Integrated Transcriptomic and Proteomic Analysis of the Pathogenic Mechanisms of *Staphylococcus aureus*-Induced Gangrenous Mastitis in Dairy Goats

**DOI:** 10.3390/ani16182878

**Published:** 2026-09-12

**Authors:** Mingzhe Fu, Xuewen Tan, Yingqiu Liu, Weimin Zhang, Shen Zhuang, Xiaopeng An, Yunpeng Fan

**Affiliations:** 1 College of Veterinary Medicine, Northwest A&F University, Yangling 712100, China; 2 Institute of Traditional Chinese Veterinary Medicine, Northwest A&F University, Yangling 712100, China; 3 College of Animal Science and Technology, Northwest A&F University, Yangling 712100, China

**Keywords:** *Staphylococcus aureus*, dairy goat, gangrenous mastitis, transcriptome, proteome

## Abstract

Gangrenous mastitis is a severe form of mastitis in dairy goats that causes extensive tissue damage and has a poor prognosis, thereby substantially affecting the dairy goat industry; however, its molecular pathogenesis remains unclear. In this study, *Staphylococcus aureus* was used to establish a model of gangrenous mastitis in dairy goats. Mammary gland tissues were collected at 72 h post-inoculation for transcriptomic and proteomic sequencing, followed by integrated multi-omics analyses to systematically identify key regulatory pathways and candidate molecules associated with *S. aureus*-induced gangrenous mastitis. The results showed that clinical mastitis was characterized mainly by activation of pathways related to the acute inflammatory response, pathogen recognition, neutrophil chemotaxis, and phagocytic defense. In contrast, gangrenous mastitis involved broader molecular reprogramming, with significant enrichment of the TNF, IL-17, and NF-κB signaling pathways, complement and coagulation cascades, platelet activation, and extracellular matrix (ECM) remodeling. Protein–protein interaction (PPI) analysis, gene set enrichment analysis (GSEA), and validation of key molecules indicated that IL6, S100A8, THBS1, SERPINE1, and MMP9 may represent important nodes in disease progression. Collectively, these findings indicate that gangrenous mastitis is a complex infectious tissue-injury process driven by inflammatory amplification, aberrant immune-cell activation, complement–coagulation dysregulation, and tissue structural disruption. The identified molecules and pathways may facilitate the development of biomarker panels for early diagnosis and risk stratification and inform preventive and adjunctive therapeutic strategies targeting excessive inflammation, microcirculatory dysfunction, and ECM damage in dairy goats.

## Simple Summary

Gangrenous mastitis is a severe udder disease in dairy goats that can cause extensive mammary tissue damage, loss of milk production, and death. However, the biological changes that cause clinical mastitis to progress to this destructive form remain unclear. This study compared mammary tissues from healthy goats and goats with clinical or gangrenous mastitis caused by *Staphylococcus aureus*. Transcriptomic analysis was used to determine which genes were activated or suppressed, whereas proteomic analysis measured changes in proteins that perform immune, metabolic, clotting, and tissue-repair functions. Combining these approaches provides a more complete picture of disease development than examining either level alone. Clinical mastitis was mainly associated with acute inflammation and antibacterial defense, whereas gangrenous mastitis showed more extensive disturbances involving inflammation, immune-cell activity, blood clotting, circulation, and tissue structure. These findings clarify the processes associated with disease worsening and identify molecular changes with potential practical value. They may support the development of earlier diagnostic methods, improve disease-severity assessment, and inform preventive or supportive treatment strategies, thereby helping veterinarians and farmers reduce animal suffering and production losses.

## 1. Introduction

Mastitis is a major constraint on dairy goat production because it reduces milk yield and quality, increases treatment and replacement costs, and compromises animal welfare [1]. A recent systematic review and meta-analysis estimated the pooled prevalence of caprine mastitis at 30.6%, with estimates of 7.5% for clinical mastitis and 31.6% for subclinical mastitis; *Staphylococcus* spp. accounted for approximately 30% of the identified pathogens, including 11% attributed to *S. aureus* [2]. Economic modeling of dairy goat udder-health deterioration showed that a 10% reduction in milk yield decreased annual revenue by approximately EUR 90 per goat, whereas combined deterioration in production and health-related traits reduced farm profitability by up to 19 percentage points [3]. Gangrenous mastitis is less frequent but has disproportionately severe consequences. A recent hospital-based study of 35 goats treated for toxic or gangrenous mastitis reported a survival-to-discharge rate of 74%, corresponding to 26% mortality [4]. Moreover, a commercial-herd outbreak of *S. aureus* mastitis caused fatal gangrenous disease in approximately 30% of the herd; *S. aureus* was isolated from 58.3% of goats in the hospital pen and from 15.0% of clinically normal mature goats, demonstrating the potential for clinically inapparent infections to sustain severe outbreaks [5]. Experimental evidence further showed that four of ten *S. aureus*-challenged goats developed gangrenous mastitis [6]. These data underscore the substantial production, economic, and animal-welfare burden of caprine mastitis and the need to clarify the mechanisms that drive progression to the gangrenous form.

Among the pathogens associated with goat mastitis, *S. aureus* is particularly important because this contagious, Gram-positive, coagulase-positive bacterium can cause persistent subclinical, clinical, and gangrenous mastitis [7]. Its adhesins and biofilm-forming ability facilitate mammary colonization and persistent infection, whereas its virulence factors contribute to immune evasion and tissue injury [8]. Moreover, *S. aureus* was identified as the causative agent in 15 goats with gangrenous mastitis [9]. Therefore, its clinical importance, broad disease spectrum, and isolation from a naturally occurring case of gangrenous mastitis justified its selection for this study.

The development of gangrenous mastitis may not simply represent a quantitative intensification of an ordinary inflammatory response, but rather the combined effects of multiple pathological processes [10]. Studies of gangrenous mastitis in dairy goats have associated severe outcomes with elevated inflammatory mediators, impaired phagocytic killing, and the actions of *S. aureus* toxins [11,12]. These observations suggest that the disease may arise through interactions between pathogen virulence and dysregulated host immunity. In other severe bacterial infections and sepsis-like inflammatory conditions, excessive inflammation can further trigger complement activation, coagulation disorders, vascular endothelial injury, microcirculatory dysfunction, and tissue hypoxia, ultimately causing structural destruction and organ dysfunction [13,14,15]. A broader analysis of gangrenous mastitis may therefore help reveal the mechanisms that drive its severe pathology.

In recent years, omics technologies have provided new approaches for investigating the pathogenesis of mastitis [16]. Transcriptome sequencing characterizes host immune and metabolic responses at the gene-expression level, whereas proteomics measures changes in protein abundance more directly and captures potential functional effectors, including inflammatory mediators, complement and coagulation proteins, ECM-related proteins, and metabolic enzymes [17]. Integrated analysis of bovine mastitis microarray and RNA-seq datasets using weighted gene co-expression network analysis and machine-learning approaches has implicated NOD-like receptor, IL-17, and TNF signaling in disease-associated inflammation [18]. Moreover, transcriptional profiling indicates that *S. aureus* induces distinct gene-expression responses in mammary epithelial cells, milk somatic cells, and peripheral immune-cell populations [19]. The resulting differentially expressed genes are primarily enriched in inflammation, immune responses, cytokine–cytokine receptor interactions, chemokine signaling, endocytosis, and pathogen recognition, indicating multilevel regulation involving the epithelial barrier, innate immunity, and immune-cell recruitment [20]. Comparative transcriptomic analysis of caprine mammary epithelial cells further suggests that different pathogens induce distinct programs involving immunity, autophagy or apoptosis, and tissue repair and remodeling [21]. Nevertheless, most previous studies have focused on clinical mastitis or early cellular responses to infection, and the systemic mechanisms underlying the onset and progression of gangrenous mastitis remain insufficiently characterized. Moreover, a single omics layer cannot fully resolve the relationship between mRNA abundance and protein abundance or function [22]. Integrated transcriptomic and proteomic analysis can identify molecules and pathways that change consistently across regulatory levels and is therefore well suited to the investigation of a complex pathological process such as gangrenous mastitis.

The objectives of this study were to compare mammary transcriptomic and proteomic profiles among control, clinical mastitis, and gangrenous mastitis goats; identify pathways and molecules associated with progression to gangrenous mastitis; and validate selected candidates using RT-qPCR and Western blotting. These candidates may subsequently be evaluated in milk or blood as minimally invasive markers for early diagnosis and severity assessment, while the identified pathways may provide targets for developing adjunctive treatments to limit irreversible mammary damage. Earlier recognition may also facilitate timely treatment, animal isolation, and herd-level prevention. Consistent with the WHO One Health framework, improved control of *S. aureus* mastitis may promote animal welfare and milk safety, reduce unnecessary antimicrobial use, and limit the spread of pathogenic or antimicrobial-resistant bacteria at the animal–human–environment interface.

## 2. Materials and Methods

### 2.1. Ethics Approval

All animal procedures performed in this experimental study were approved by the Animal Ethics and Welfare Committee of Northwest A&F University (approval no. XN2023-1111; approval date: [26 September 2023]) and were conducted in accordance with the relevant national guidelines of China.

### 2.2. Isolation, Identification, and Whole-Genome Characterization of the S. aureus Strain

Milk samples were aseptically collected from dairy goats with naturally occurring gangrenous mastitis at a goat farm in Yangling District, Shaanxi Province. The samples were streaked directly onto blood agar plates and incubated aerobically at 37 °C for 24 h, as described previously [23]. A representative colony was selected and restreaked onto fresh blood agar five successive times. After the final subculture, culture purity was assessed based on uniform colony morphology and Gram staining. A single colony was inoculated into Tryptic Soy Broth (TSB), and genomic DNA was extracted from the resulting culture. The 16S rRNA gene was amplified and Sanger-sequenced using a 3730XL Genetic Analyzer (Applied Biosystems, Foster City, CA, USA). After quality trimming, the sequence was analyzed using NCBI BLASTn (https://blast.ncbi.nlm.nih.gov; accessed on 11 March 2023) and showed 100% identity to *S. aureus* strain NCTC 9555 (GenBank accession no. LR134090.1). Whole-genome sequencing and bioinformatic analysis of the challenge isolate were performed by Novogene Co., Ltd. (Beijing, China). Raw reads were quality-filtered using fastp [24]. Clean reads were assembled using SOAPdenovo (v2.04), SPAdes (v3.15.5), and ABySS (v2.3.7), and the assemblies were integrated using CISA (v1.3) and refined using GapCloser (v1.12); sequences shorter than 500 bp were excluded. Protein-coding genes were predicted using GeneMarkS (v4.17). The predicted protein sequences were aligned against the Virulence Factor Database (VFDB) using DIAMOND (v2.1.8) with an E-value threshold of 1 × 10^−5^. For the curated presentation of major virulence determinants, matches with amino acid identity ≥ 80% and query coverage ≥ 65% were classified as high-confidence, whereas matches with 60–79.9% identity and ≥60% query coverage were classified as moderate-confidence.

### 2.3. Experimental Animals

Nine healthy lactating Guanzhong dairy goats, aged 1.5 years and weighing 40 ± 2.5 kg, were maintained under identical environmental conditions with ad libitum access to feed and water. Before the experiment, all goats underwent health examinations to confirm the absence of overt mammary abnormalities. Animals were randomly assigned to the control (CON), clinical mastitis (CLM), or gangrenous mastitis (GSM) group, with three goats per group.

### 2.4. Control Material, Control Animals, and Sham-Control Procedures

Sterile phosphate-buffered saline (PBS, BL2220A, Biosharp, Beijing, China) was used both to prepare the bacterial suspensions and as the vehicle control. The CON group consisted of three goats meeting the same health and inclusion criteria described above. After teat-end preparation using 75% ethanol, each control goat received 3 mL of sterile PBS through the teat canal using a sterile syringe, followed by gentle mammary massage for 10 s. No bacteria were included in the control infusion material. The control goats underwent the same restraint, post-infusion handling, 72-h clinical observation, euthanasia, mammary-tissue collection, and sample-processing procedures as the CLM and GSM goats. Samples were collected from corresponding anatomical regions of the mammary gland in all groups. Therefore, the control procedure accounted for the potential effects of animal handling, intramammary infusion, the PBS vehicle, observation time, and tissue collection.

### 2.5. S. aureus Challenge and Model Assessment

Mastitis of different severities was induced in the CLM and GSM groups by intramammary infusion of *S. aureus* through the teat canal. After recovery, culture, and enumeration, the bacterial suspension was adjusted to the required concentration using sterile PBS. The challenge concentrations and the 72-h terminal endpoint were selected based on a preliminary dose-ranging experiment conducted in accordance with the ethical approval for the present study using the same *S. aureus* isolate. In this preliminary experiment, concentrations of 1 × 10^5^ and 1 × 10^8^ CFU/mL produced clearly distinguishable clinical and gangrenous mastitis phenotypes, respectively, by 72 h post-inoculation. Accordingly, the CLM and GSM groups received 3 mL of bacterial suspensions containing 1 × 10^5^ and 1 × 10^8^ CFU/mL, respectively, through the teat canal, followed by gentle mammary massage for 10 s. The same experimental cohort and challenge model were previously reported in our study [25]. Disease phenotypes were evaluated at 72 h post-inoculation using the clinical scoring framework described by Rainard et al. [6]. Clinical assessment included body temperature, general condition, milk appearance, local mammary signs on palpation, and mammary skin color. Clinical mastitis was defined by abnormal milk, including flakes or clots, together with mammary swelling, firmness, and tenderness, but without skin discoloration or tissue necrosis. Gangrenous mastitis was distinguished by severe systemic illness and characteristic mammary changes, including pronounced swelling and hardening, purplish-red to dark skin discoloration, reddish mammary secretion, cessation of milk production, and visible tissue necrosis. At 72 h post-inoculation, the goats were euthanized by intravenous administration of an overdose of pentobarbital sodium; thus, they were not treated, returned to milk production, or allowed to enter the food chain. The mammary glands were then aseptically exposed, and glandular parenchymal tissue samples were collected using sterile instruments according to a previously described postmortem sampling procedure [26]. Samples were obtained from corresponding anatomical regions of the mammary gland in all animals while avoiding the skin and external adipose tissue. The tissue samples were immediately divided into aliquots, snap-frozen in liquid nitrogen, and stored at −80 °C until RNA and protein extraction. Following tissue collection, the carcasses and remaining biological materials were disposed of in accordance with institutional biosafety and animal-waste disposal requirements.

### 2.6. Transcriptome Sequencing

Total RNA was isolated from mammary gland samples with TRIzol reagent (15596018CN, Invitrogen, Carlsbad, CA, USA). RNA quality was evaluated by measuring purity with a NanoDrop spectrophotometer (NanoDrop 2000, Thermo, Waltham, MA, USA), concentration with a Qubit fluorometer, and integrity with a Bioanalyzer (2100, Agilent, Santa Clara, CA, USA). Only qualified RNA samples were used to construct sequencing libraries. Poly(A)-enriched mRNA was fragmented and converted into cDNA, followed by end repair, adaptor attachment, and PCR enrichment. The prepared libraries were subsequently sequenced using the Illumina NovaSeq 6000 platform. Raw sequencing data were filtered with fastp (v0.20.1) to eliminate adaptor-containing sequences, low-quality reads, and reads with excessive ambiguous bases. The retained reads were mapped against the *Capra hircus* ARS1.2 reference genome using HISAT2 (v2.1.0). Read counts at the gene level were obtained with featureCounts (v2.0.1) according to NCBI RefSeq *Capra hircus* Annotation Release 102, while gene abundance was expressed as fragments per kilobase of transcript per million mapped reads (FPKM). Differentially expressed genes were identified using edgeR (v3.40.2), with *p*-values adjusted by the Benjamini–Hochberg method. Genes meeting a false discovery rate (FDR; q-value) < 0.05 and |log_2_ fold change (FC)| > 1 were regarded as differentially expressed.

### 2.7. Proteomics Sequencing

Mammary gland tissues were homogenized thoroughly in protein lysis buffer (50 mM Tris, pH 8.1, and 1% SDS), and total protein was recovered. Protein content was quantified using a bicinchoninic acid (BCA) assay (23225, Thermo, Waltham, MA, USA), whereas sample quality was examined by sodium dodecyl sulfate–polyacrylamide gel electrophoresis (SDS-PAGE). Equivalent protein aliquots were treated with 5 mM dithiothreitol for 30 min at 55 °C and subsequently incubated with 10 mM iodoacetamide for 15 min under light-protected conditions. Samples were transferred into 200 mM triethylammonium bicarbonate buffer and digested with trypsin at 37 °C for 12 h. Peptides were derivatized with tandem mass tag (TMT) reagents following the manufacturer’s protocol. The labeled samples were then combined, separated by high-pH reversed-phase high-performance liquid chromatography, and subjected to liquid chromatography–tandem mass spectrometry. Raw spectra were searched, and reporter-ion intensities were quantified using Proteome Discoverer (v2.4.1.15) against the NCBI RefSeq *Capra hircus* protein database corresponding to Annotation Release 102. For database interrogation, trypsin was selected as the protease, with a maximum of two missed cleavages permitted. Cysteine carbamidomethylation was defined as a fixed modification, whereas methionine oxidation and the relevant TMT labeling were treated as variable modifications. A target–decoy strategy was used, and peptide-spectrum matches and protein identifications were filtered to an FDR < 1%. Protein abundance was normalized using reporter-ion intensities. Differentially expressed proteins were screened using the prespecified criteria of *p* < 0.05 and |log_2_FC| > 1; Benjamini–Hochberg-adjusted *p*-values were additionally calculated to evaluate the robustness of the discovery results.

### 2.8. GO and KEGG Enrichment Analyses

Differentially expressed genes and proteins were annotated separately using the GO and KEGG databases. GO terms were evaluated within the biological process, cellular component, and molecular function categories, whereas KEGG analysis was used to identify the principal signaling and metabolic pathways represented by the differential molecules. Statistical enrichment was evaluated by the hypergeometric test, with the resulting *p*-values adjusted for multiple testing according to the Benjamini–Hochberg method. Terms or pathways with an adjusted *p*-value < 0.05 were considered significantly enriched; nominal *p*-values were retained in the complete enrichment output for transparent reporting.

### 2.9. Transcriptome–Proteome Integration Workflow

The experimental cohort and the generation of the underlying transcriptomic and proteomic datasets have been reported previously [25]. For the present analysis, transcriptomic and proteomic datasets generated from the same mammary gland samples were integrated by mapping transcript and protein identifiers to common gene symbols using the NCBI RefSeq database (*Capra hircus* Annotation Release 102; accessed 15 January 2024). For each group comparison, RNA- and protein-level log_2_FC values were matched and visualized in association scatter plots. Molecules were classified as concordantly upregulated, concordantly downregulated, discordantly regulated, RNA-specific, or protein-specific according to their significance and direction of change at the two levels. Concordantly regulated molecules were prioritized for biological interpretation and were subjected to integrated GO and KEGG enrichment, PPI-network, and GSEA analyses. This workflow allowed both agreement and divergence between transcriptional and protein-level regulation to be assessed explicitly.

### 2.10. Protein–Protein Interaction Network Analysis

Concordantly regulated molecules were submitted to the STRING database for PPI-network analysis, and the resulting networks were visualized using Cytoscape (v3.7.1). Hub nodes were ranked according to degree centrality, after which candidate key molecules were selected by jointly considering network connectivity, direction of expression change, and biological function.

### 2.11. Gene Set Enrichment Analysis

To evaluate coordinated pathway-level changes without restricting the analysis to molecules passing a differential-expression cutoff, all quantified genes and proteins were ranked separately for each comparison according to their expression-change statistics. KEGG gene sets were then analyzed by GSEA at the transcript and protein levels, and normalized enrichment scores (NESs) were compared in the integrated analysis. Statistical significance was determined from permutation-derived *p*-values with Benjamini–Hochberg correction. In the figures, asterisks denote pathways meeting the prespecified significance criterion.

### 2.12. RT-qPCR

To validate the transcriptomic results, the key candidate genes IL6, PTGS2, S100A8, THBS1, SERPINE1, and MMP9 were selected for quantitative reverse-transcription PCR (RT-qPCR). Primer pairs were designed with NCBI tools and produced by Sangon Biotech (Shanghai, China), with their sequences provided in Table 1. Following quality evaluation, total RNA isolated from mammary gland tissues with TRIzol reagent was converted to cDNA using the PrimeScript RT Reagent Kit with gDNA Eraser (DN2212-01, DiNing, Beijing, China). Amplification was conducted with SYBR Green chemistry (DN2055-05, DiNing, Beijing, China), beginning with denaturation at 95 °C for 30 s and continuing for 40 cycles of 95 °C for 5 s and 60 °C for 30 s. Three technical reactions were performed for each sample. β-actin served as the internal reference, and relative transcript abundance was determined by the 2^−ΔΔCt^ method.

### 2.13. Western Blotting

To validate the proteomic results, IL6, PTGS2, S100A8, THBS1, SERPINE1, and MMP9 were selected for Western blot analysis. Mammary gland samples were lysed in radioimmunoprecipitation assay buffer (P0013G, Beyotime, Shanghai, China), and the resulting protein content was quantified by the BCA method. Equivalent protein quantities were resolved by SDS-PAGE and electrotransferred to polyvinylidene fluoride membranes (IPVH00010, Merck, Darmstadt, Germany). Following blocking, the membranes were exposed to the appropriate primary antibodies at 4 °C overnight. After washing, they were treated with horseradish peroxidase-linked secondary antibodies at room temperature. Immunoreactive bands were detected by enhanced chemiluminescence and recorded using an imaging system. β-actin was employed as the loading reference, and densitometric analysis was conducted with ImageJ (v1.54s). Details of the antibodies, including their suppliers and catalog numbers, are presented in Table 2.

### 2.14. Statistical Analysis

Statistical analyses were performed using R (v4.2.2) and GraphPad Prism (v10.0). Data are presented as the mean ± standard deviation from three biological replicates per group. Differences among groups were assessed by one-way analysis of variance. A *p*-value < 0.05 was considered statistically significant.

## 3. Results

### 3.1. Whole-Genome Characterization of the S. aureus Strain

Whole-genome sequencing generated 1049.30 Mb of clean data, with Q20 and Q30 values of 99.52% and 97.70%, respectively. The draft genome comprised 51 contigs (>500 bp), with a total length of 2,795,074 bp, an N50 of 112,623 bp, a GC content of 32.8%, and 2661 predicted protein-coding genes (Table S1). High-confidence VFDB matches included hemolysin genes (*hla*, *hlb*, and *hlgABC*), leukocidin genes encoding LukMF′, LukGH, and LukED, the superantigen genes tst, sec, and selL, and genes associated with adhesion, biofilm formation, capsule production, proteolysis, iron acquisition, and type VII secretion. Matches to coa and vWbp were retained as putative moderate-confidence findings (Table S2).

### 3.2. Transcriptomic Differential-Expression and Functional-Enrichment Analyses

**Figure 1:**
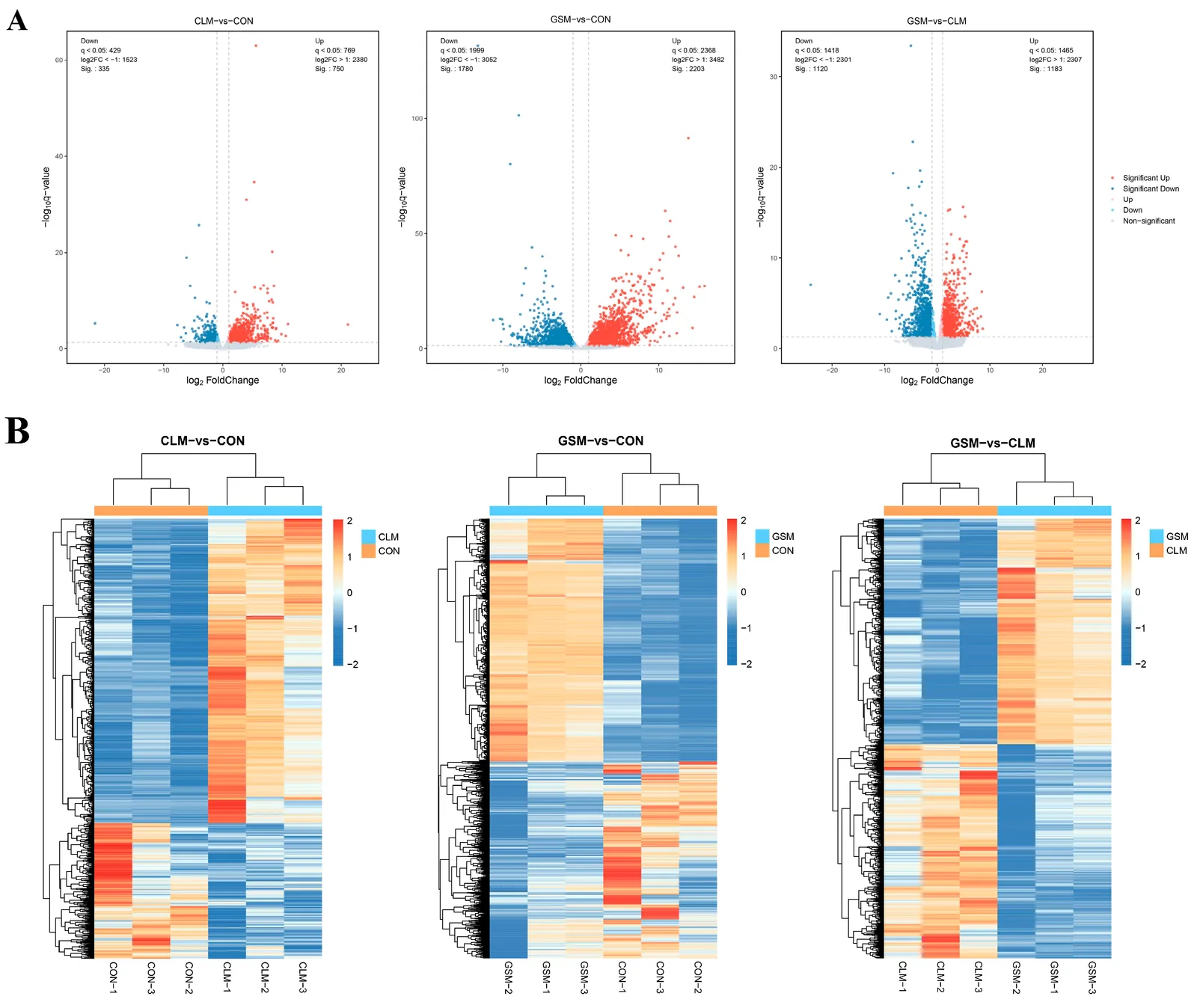

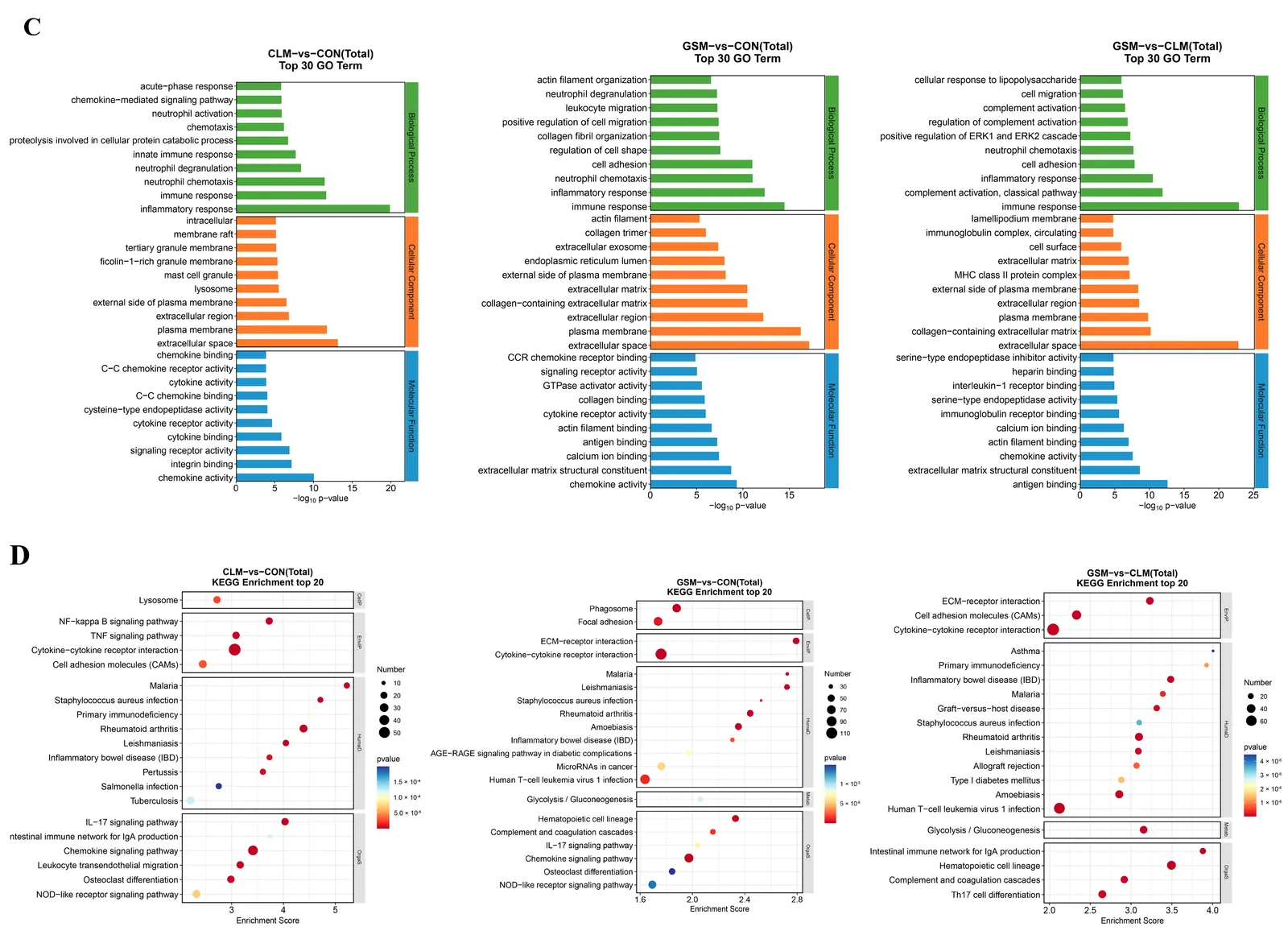
Transcriptomic profiling of mammary gland tissues from control, clinical mastitis and gangrenous mastitis groups induced by *S. aureus*. (**A**) Volcano plot of differentially expressed genes. (**B**) Hierarchical clustering heatmap of differentially expressed genes. (**C**) GO enrichment analysis of differentially expressed genes. (**D**) KEGG pathway enrichment analysis of differentially expressed genes.

To characterize transcriptomic changes in mammary gland tissues across pathological states, differential-expression analyses were performed among the CON, CLM, and GSM groups. PCA showed separation among the CON, CLM, and GSM groups, with biological replicates generally clustering within their respective groups (Figure S1). Using q-value < 0.05 and |log_2_FC| > 1 as the screening criteria, 1085 differentially expressed genes (DEGs) were identified in CLM versus CON, including 750 upregulated and 335 downregulated genes. A total of 3983 DEGs were identified in GSM versus CON, including 2203 upregulated and 1780 downregulated genes, whereas 2303 DEGs were identified in GSM versus CLM, including 1183 upregulated and 1120 downregulated genes. The markedly larger number of DEGs in GSM than in CLM indicated more extensive transcriptional reprogramming in the mammary gland during gangrenous mastitis (Figure 1A). Hierarchical-clustering heatmaps revealed distinct differential-expression patterns in all three comparisons, and samples from the different groups were readily separated according to DEG expression profiles (Figure 1B).

To investigate the potential biological functions of the DEGs, GO and KEGG enrichment analyses were performed for all three comparisons. In the GO analysis, DEGs in CLM versus CON were primarily enriched in inflammatory responses, immune responses, and neutrophil chemotaxis and activation. In addition to these processes, DEGs in GSM versus CON were significantly enriched in leukocyte migration, cell adhesion, and ECM remodeling, whereas those in GSM versus CLM showed further enrichment in complement activation, inflammatory responses, cell migration, and immune responses (Figure 1C). KEGG analysis showed that DEGs in CLM versus CON were mainly enriched in the NF-κB, TNF, IL-17, chemokine, and NOD-like receptor signaling pathways. DEGs in GSM versus CON showed additional enrichment in complement and coagulation cascades, ECM–receptor interaction, phagosome, and cytokine–cytokine receptor interaction, whereas those in GSM versus CLM were primarily enriched in cytokine–cytokine receptor interaction, complement and coagulation cascades, Th17-cell differentiation, hematopoietic cell lineage, and ECM–receptor interaction (Figure 1D). These findings indicate that gangrenous mastitis is characterized by stronger inflammatory responses, complement activation, and tissue remodeling than clinical mastitis.

### 3.3. Proteomic Differential-Expression and Functional-Enrichment Analyses

**Figure 2:**
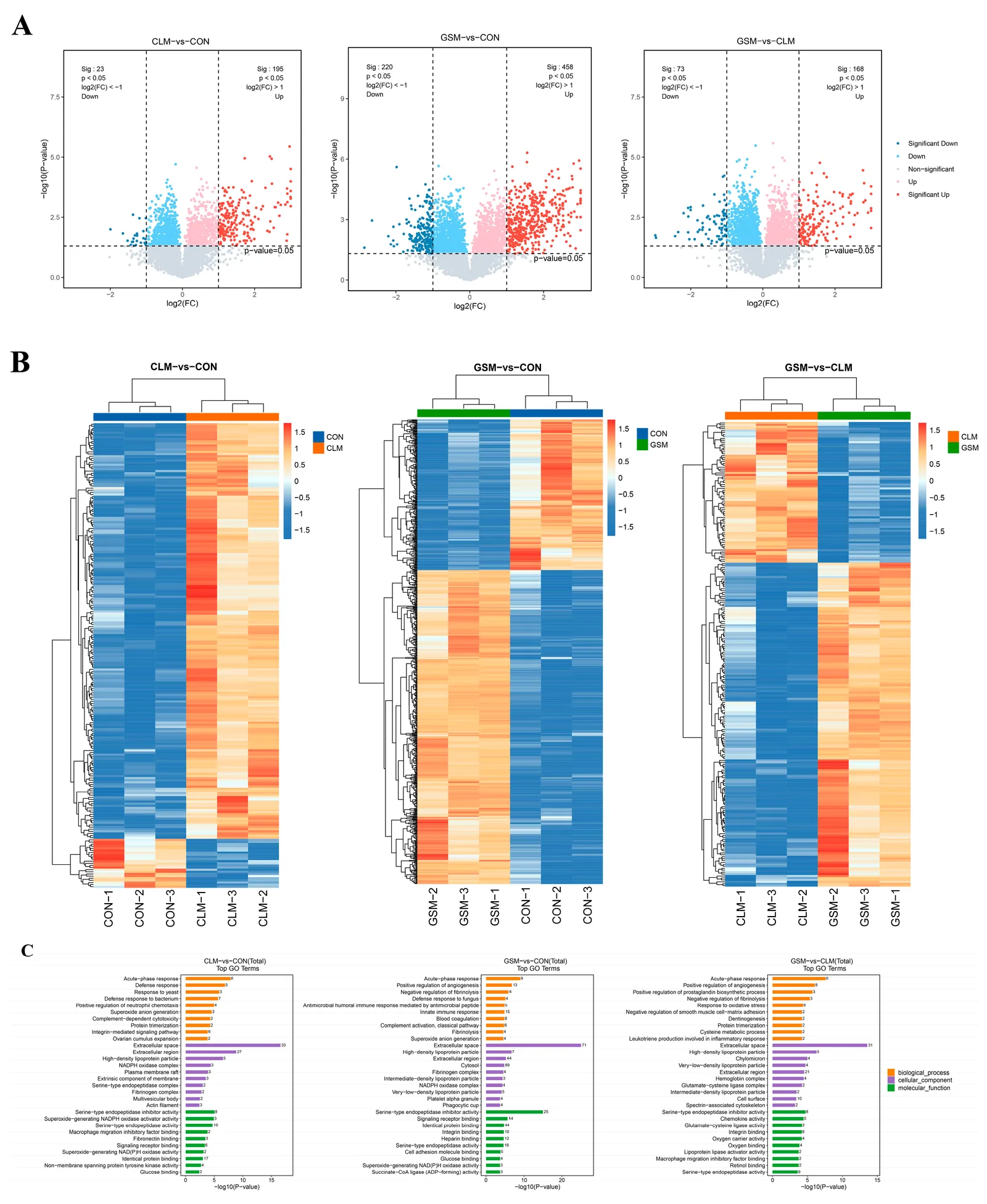

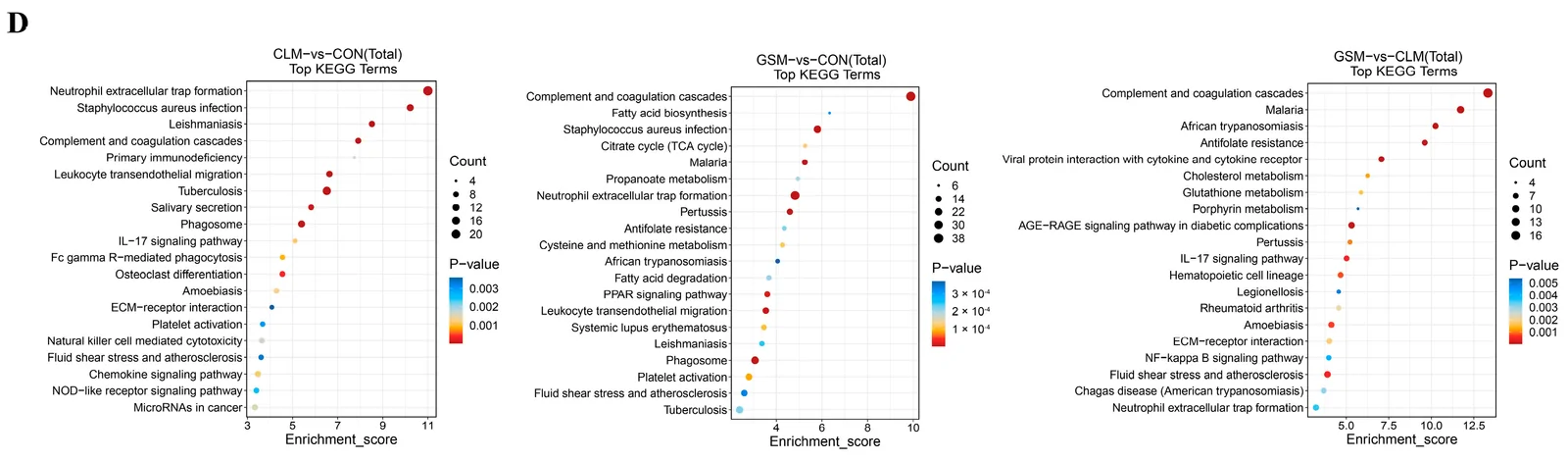
Proteome profiling of mammary gland tissues from control, clinical mastitis and gangrenous mastitis groups induced by *S. aureus*. (**A**) Volcano plot of differentially expressed proteins. (**B**) Hierarchical clustering heatmap of differentially expressed proteins. (**C**) GO enrichment analysis of differentially expressed proteins. (**D**) KEGG pathway enrichment analysis of differentially expressed proteins.

To further characterize protein-level changes in mammary gland tissues across mastitis states, proteomic differential-expression analyses were performed among the CON, CLM, and GSM groups. A total of 6079 protein groups were identified at an FDR of <1%. Using *p* < 0.05 and |log_2_FC| > 1 as the screening criteria, 218 differentially expressed proteins (DEPs) were identified in CLM versus CON, including 195 upregulated and 23 downregulated proteins. A total of 678 DEPs were identified in GSM versus CON, including 458 upregulated and 220 downregulated proteins, whereas 241 DEPs were identified in GSM versus CLM, including 168 upregulated and 73 downregulated proteins. The substantially larger number of DEPs in GSM than in CLM indicated more pronounced protein-expression changes in the mammary gland during gangrenous mastitis (Figure 2A). Hierarchical-clustering heatmaps showed clear DEP patterns in all three comparisons, and samples from different groups were readily distinguished by their protein-expression profiles (Figure 2B).

GO analysis showed that DEPs in CLM versus CON were mainly enriched in the acute-phase response, defense response, neutrophil chemotaxis, and superoxide-anion generation. DEPs in GSM versus CON showed further enrichment in blood coagulation, complement activation, regulation of fibrinolysis, innate immune responses, and angiogenesis, whereas DEPs in GSM versus CLM were primarily enriched in the acute-phase response, oxidative stress, negative regulation of fibrinolysis, and inflammatory responses (Figure 2C). KEGG analysis indicated that DEPs in CLM versus CON were mainly enriched in *S. aureus* infection, neutrophil extracellular trap formation, complement and coagulation cascades, leukocyte transendothelial migration, phagosome, and the IL-17 signaling pathway. DEPs in GSM versus CON and GSM versus CLM showed additional enrichment in complement and coagulation cascades, the NF-κB signaling pathway, ECM–receptor interaction, and metabolism-related pathways (Figure 2D). These results indicate that gangrenous mastitis is associated with more pronounced complement–coagulation activation, inflammatory amplification, oxidative stress, and tissue injury than clinical mastitis.

### 3.4. Integrated Transcriptomic and Proteomic Analysis

**Figure 3:**
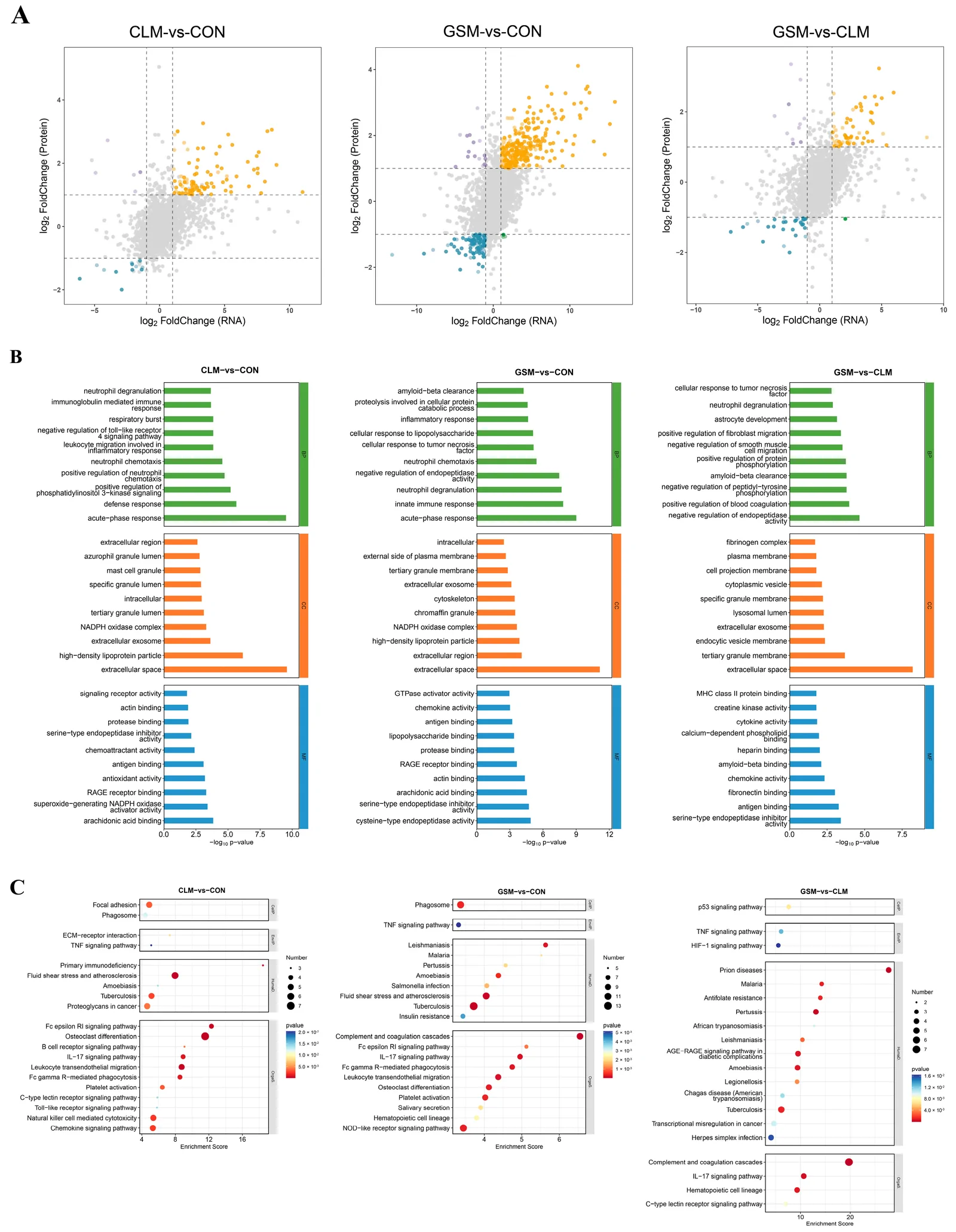
Integrated analysis of transcriptome and proteome. (**A**) Transcriptome–Proteome association scatter plot. (**B**) GO enrichment analysis of combined differential molecules. (**C**) KEGG enrichment analysis of combined differential molecules.

To identify key molecules showing concordant changes at both the mRNA and protein levels, the transcriptomic and proteomic results were integrated. Association scatter plots identified 73 concordantly upregulated and 7 concordantly downregulated molecules in CLM versus CON, 211 concordantly upregulated and 70 concordantly downregulated molecules in GSM versus CON, and 40 concordantly upregulated and 22 concordantly downregulated molecules in GSM versus CLM (Figure 3A). A quantitative summary of the transcriptomic, proteomic, and concordant differential-expression results is presented in Table 3.

GO analysis showed that the integrated differential molecules in CLM versus CON were primarily enriched in the acute-phase response, defense response, neutrophil chemotaxis, and degranulation. Those in GSM versus CON showed additional enrichment in innate immune responses, inflammatory responses, cellular responses to lipopolysaccharide and TNF, and neutrophil activation, whereas those in GSM versus CLM were mainly enriched in cellular responses to TNF, neutrophil degranulation, positive regulation of blood coagulation, and tissue remodeling (Figure 3B). KEGG analysis indicated that integrated differential molecules in CLM versus CON were primarily enriched in phagosome, TNF signaling, IL-17 signaling, leukocyte transendothelial migration, Toll-like receptor signaling, and chemokine signaling. Those in GSM versus CON showed further enrichment in complement and coagulation cascades, Fcγ receptor-mediated phagocytosis, platelet activation, and NOD-like receptor signaling, whereas those in GSM versus CLM were mainly enriched in the p53, TNF, and HIF-1 signaling pathways, complement and coagulation cascades, and IL-17 signaling (Figure 3C). These findings suggest that gangrenous mastitis involves more pronounced inflammatory amplification, complement–coagulation activation, hypoxic responses, and tissue injury than clinical mastitis.

### 3.5. PPI-Network Analysis of Concordantly Regulated Molecules

**Figure 4:**
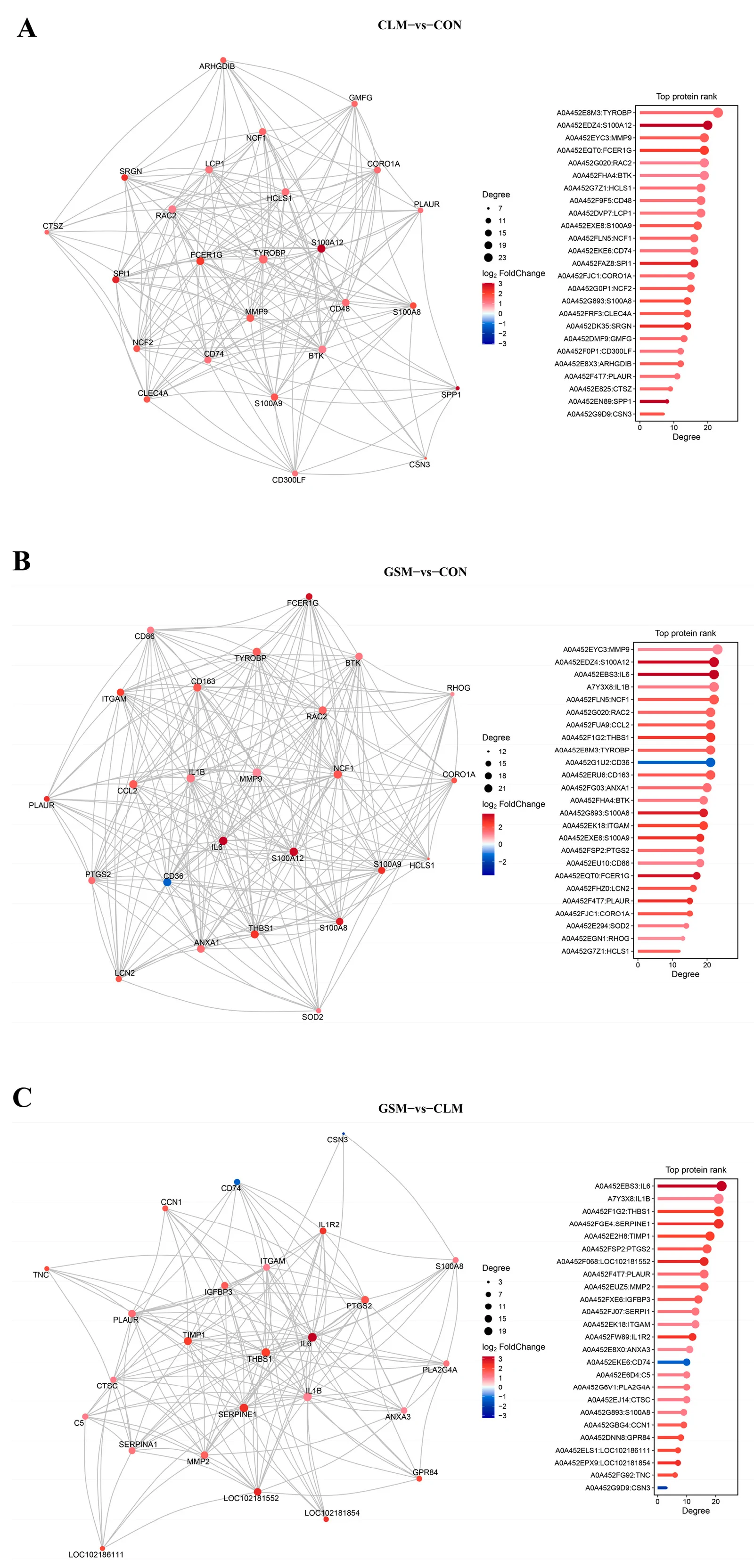
PPI network analysis of combined differential molecules. (**A**) CLM-vs.-CON PPI network. (**B**) GSM-vs.-CON PPI network. (**C**) GSM-vs.-CLM PPI network.

To identify key molecules involved in mastitis progression, PPI networks were constructed for molecules that were differentially expressed at both the transcript and protein levels. In the CLM-versus-CON network, TYROBP, S100A12, MMP9, FCER1G, RAC2, BTK, S100A8, S100A9, and NCF1 were prominent nodes, indicating that clinical mastitis predominantly activated neutrophil responses, phagocytosis, and oxidative-burst processes (Figure 4A). In the GSM-versus-CON network, MMP9, S100A12, IL6, IL1B, NCF1, RAC2, CCL2, THBS1, TYROBP, S100A8, and S100A9 occupied central positions, indicating further enhancement of inflammatory responses, chemokine signaling, neutrophil activation, and matrix injury during gangrenous mastitis (Figure 4B). In the GSM-versus-CLM network, IL6, IL1B, SERPINE1, THBS1, TIMP1, PTGS2, MMP2, PLAUR, C5, and S100A8 were key nodes, suggesting that inflammatory amplification, complement activation, coagulation–fibrinolysis dysregulation, and ECM remodeling may be important mechanisms underlying the progression of gangrenous mastitis (Figure 4C).

### 3.6. Integrated Transcriptomic and Proteomic GSEA

**Figure 5:**
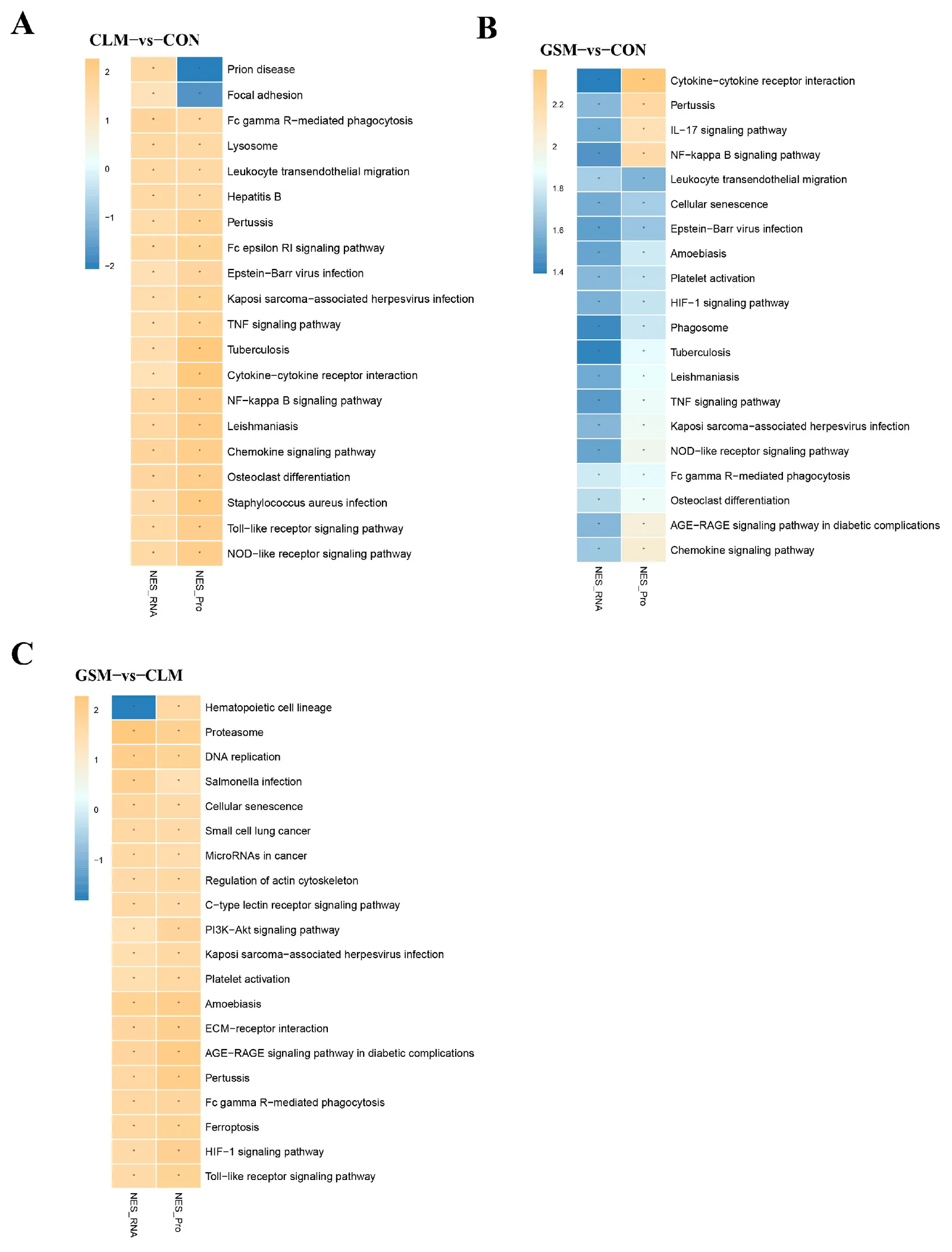
Integrated GSEA of transcriptome and proteome. (**A**) CLM-vs.-CON; (**B**) GSM-vs.-CON; (**C**) GSM-vs.-CLM. NES_RNA represents the normalized enrichment score at the RNA level, and NES_Pro represents the normalized enrichment score at the protein level. Asterisks indicate significantly enriched pathways.

To further assess coordinated changes in key pathways at both the transcript and protein levels, integrated GSEA was conducted for the CON, CLM, and GSM groups. In CLM versus CON, enriched pathways included *S. aureus* infection, Toll-like receptor signaling, NOD-like receptor signaling, NF-κB signaling, TNF signaling, chemokine signaling, leukocyte transendothelial migration, Fcγ receptor-mediated phagocytosis, and lysosome, indicating that pathogen recognition, inflammatory responses, and phagocytic defense were the predominant processes activated during clinical mastitis (Figure 5A). In GSM versus CON, the IL-17, NF-κB, TNF, NOD-like receptor, chemokine, and HIF-1 signaling pathways, together with phagosome, leukocyte transendothelial migration, and platelet activation, were significantly enriched, indicating stronger inflammatory amplification, immune-cell migration, phagocytic responses, and hypoxic adaptation during gangrenous mastitis (Figure 5B). In GSM versus CLM, enrichment was observed in platelet activation, ECM–receptor interaction, HIF-1 signaling, cellular senescence, PI3K–Akt signaling, and hematopoietic cell lineage, suggesting that these processes may contribute to the progression of gangrenous mastitis (Figure 5C). The principal pathways and representative molecules identified by the integrated transcriptomic and proteomic analyses are summarized in Table 4.

### 3.7. Validation of Key Differentially Expressed Genes and Proteins

**Figure 6:**
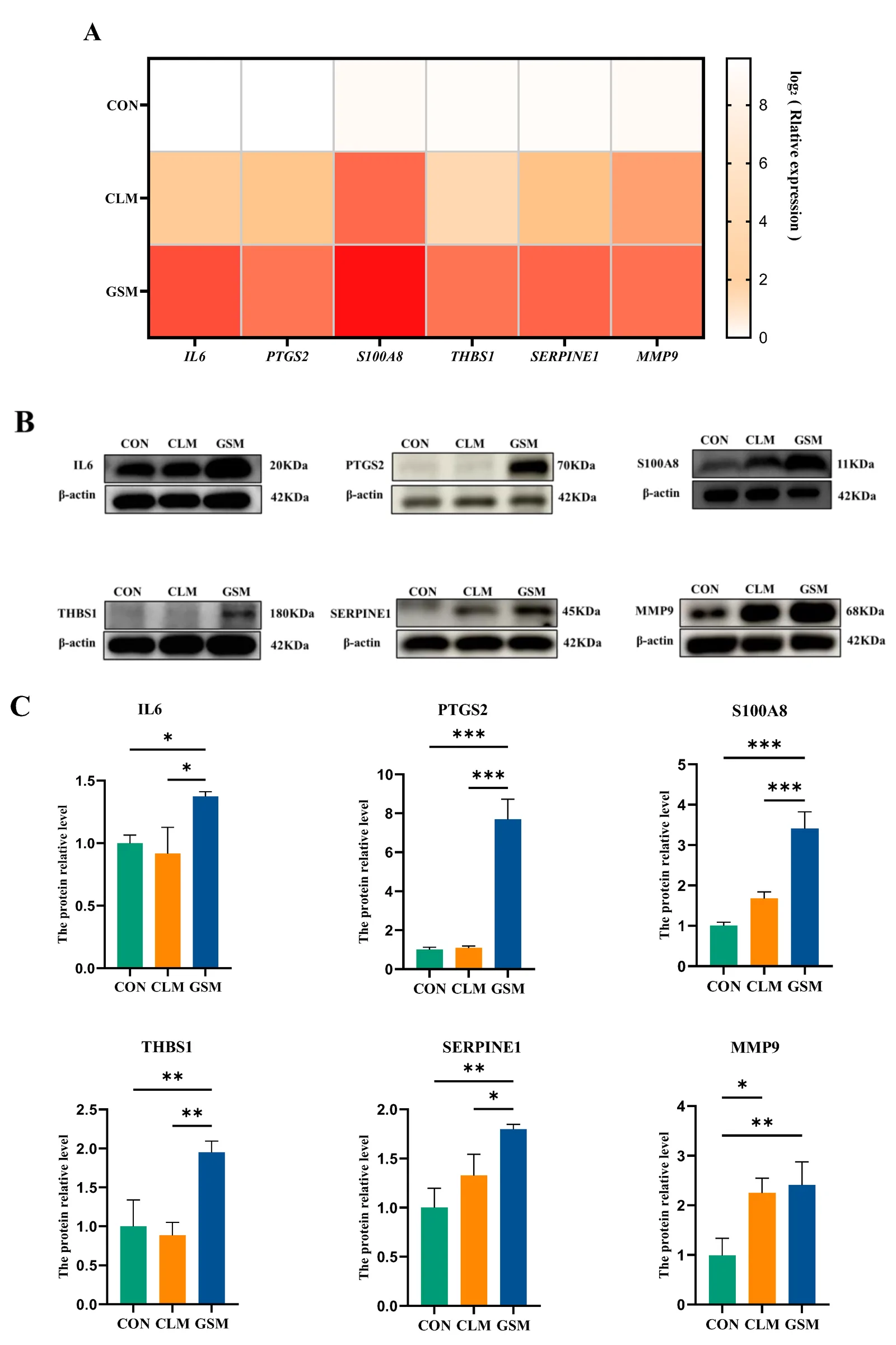
Validation of key differentially expressed genes and proteins. (**A**) The mRNA relative expression of *IL6*, *PTGS2*, *S100A8*, *THBS1*, *SERPINE1*, and *MMP9*. (**B**) Protein bands. (**C**) The protein relative expression of IL6, PTGS2, S100A8, THBS1, SERPINE1, and MMP9. Data are presented as the mean ± SD, *n* = 3. * *p* < 0.05, ** *p* < 0.01, *** *p* < 0.001.

To evaluate the reliability of the multi-omics results, the key candidate molecules IL6, PTGS2, S100A8, THBS1, SERPINE1, and MMP9 were selected for RT-qPCR and Western blot validation. Their expression patterns were generally consistent with the transcriptomic and proteomic results, further supporting their potential roles in the progression of *S. aureus*-induced gangrenous mastitis in dairy goats (Figure 6).

## 4. Discussion

Using integrated transcriptomic and proteomic analyses, we compared mammary-gland expression profiles among the CON, CLM, and GSM groups. Both mastitis groups showed pronounced immune and inflammatory changes, whereas GSM exhibited more DEGs and DEPs and broader pathway enrichment. Compared with CLM, GSM showed stronger enrichment of terms related to neutrophil activation, complement–coagulation dysregulation, platelet activation, HIF-1-associated hypoxic responses, ECM remodeling, and cellular injury. The RT-qPCR and Western blot results for IL6, PTGS2, S100A8, THBS1, SERPINE1, and MMP9 were generally consistent with the omics findings. Because enrichment analysis, PPI connectivity, and expression validation identify associations rather than pathway activation or causality, the following interpretations should be considered biologically supported hypotheses requiring functional verification.

Following *S. aureus* infection, host pattern-recognition receptors detect bacterial components and activate the NF-κB, TNF, IL-17, NOD-like receptor, and chemokine signaling pathways. Enrichment of these pathways in both CLM versus CON and GSM versus CON was consistent with prominent involvement of innate immune responses. However, the greater enrichment of TNF and IL-17 signaling and the central positions of IL6, IL1B, and PTGS2 in the GSM PPI networks were consistent with stronger inflammatory signaling in GSM. Excessive inflammatory mediators can promote leukocyte recruitment, increase vascular permeability, and disturb complement, coagulation, and hypoxia-related responses during severe bacterial infection [27,28,29]. IL6 promotes local inflammatory cascades, whereas PTGS2-dependent prostaglandin synthesis can amplify inflammation and tissue injury [30]. In cattle, *S. aureus*-derived lipoteichoic acid increases IL6 expression and secretion in mammary epithelial cells, and PTGS2 has been associated with inflammatory stress, apoptosis, and mammary-tissue damage [31]. These cross-species findings support the inflammatory relevance of IL6 and PTGS2 but do not demonstrate that either gene drives progression to gangrenous mastitis in goats. Functional perturbation and pathway-activity assays are required to test their causal roles.

Neutrophils eliminate *S. aureus* through phagocytosis, oxidative burst, degranulation, and neutrophil extracellular trap formation [32], but excessive activation can also release reactive oxygen species and proteases that damage host tissues [33]. Transcriptomic, proteomic, and integrated analyses in the present study consistently showed significant enrichment of neutrophil chemotaxis, neutrophil degranulation, respiratory burst, NADPH oxidase complex, and NET-formation processes, supporting a strong association between neutrophil-related responses and mammary injury. S100A8, S100A9, S100A12, NCF1, NCF2, RAC2, MMP9, and ITGAM repeatedly appeared in the PPI networks. S100A8, S100A9, and S100A12 are closely associated with myeloid-cell activation and acute inflammation [34]; NCF1, NCF2, and RAC2 contribute to NADPH oxidase assembly and oxidative burst [35,36]; ITGAM mediates myeloid-cell adhesion and migration [37]; and MMP9 promotes matrix degradation and structural tissue damage [38]. In septic mice, S100A9 knockout, which eliminated the functional S100A8/A9 complex, reduced pulmonary inflammatory cytokine expression, vascular leakage, and lung injury [39]. In an LPS-induced acute lung injury model, exogenous MMP9 increased inflammatory responses and endothelial permeability in murine pulmonary microvascular endothelial cells, while MMP9 knockdown produced the opposite effects [40]. These findings suggest that excessive S100A8/A9 and MMP9 activity can aggravate inflammation and tissue-barrier damage in other species. Therefore, neutrophil responses may support pathogen clearance during clinical mastitis but contribute to tissue injury when excessively activated during gangrenous mastitis, as reported in other severe inflammatory conditions [41,42,43]. The validation of S100A8 and MMP9 confirms expression consistency but does not establish their cellular sources or functions in goat mammary tissue; direct assays of neutrophil activity, NET formation, and MMP9 enzymatic activity remain necessary.

Complement and coagulation cascades were consistently enriched in GSM versus CON and GSM versus CLM. Moreover, THBS1, SERPINE1, PLAUR, and C5 occupied important positions in the GSM-versus-CLM PPI network, suggesting that complement–coagulation dysregulation is associated with disease severity. Although moderate complement activation supports pathogen clearance, excessive activation can increase leukocyte recruitment, vascular permeability, and tissue damage [44]. Its interaction with coagulation may further amplify inflammation and microcirculatory dysfunction [45]. The validated increase in SERPINE1 is compatible with a hypofibrinolytic and prothrombotic state [46]. Whereas THBS1 and PLAUR may connect platelet and vascular responses with fibrinolysis and tissue remodeling [47,48]. In human THP-1 monocytes, THBS1 silencing decreased LPS-induced IL-6, IL-1β, and TNF-α secretion, while THBS1 overexpression produced the opposite effects through NF-κB signaling [49]. In mice with DSS-induced colitis, PAI-1 deficiency reduced NF-κB activation, proinflammatory cytokine expression, neutrophil infiltration, and epithelial injury [50]. These findings suggest that increased THBS1 and SERPINE1 expression may aggravate inflammatory tissue injury in other species, although their functions in the caprine mammary gland require direct verification. Together, these alterations may promote local microcirculatory impairment and ischemic tissue injury during gangrenous mastitis.

The structural integrity of mammary gland tissue depends on the stability of the ECM, cell-adhesion molecules, and the epithelial barrier [51]. The enrichment of ECM–receptor interaction, focal adhesion, and matrix-organization terms, together with the prominent positions of MMP2, MMP9, TIMP1, THBS1, and PLAUR in the PPI networks, was consistent with ECM-remodeling-associated expression changes in gangrenous mastitis. MMP2 and MMP9 degrade multiple ECM components [52,53]. Changes in TIMP1, a major endogenous inhibitor of matrix metalloproteinases, suggest disruption of the balance between extracellular matrix degradation and its inhibition [54]. THBS1 and PLAUR may additionally regulate cell–matrix interactions, fibrinolysis, and inflammatory-cell migration [55]. Similar dysregulated matrix-remodeling processes can disrupt tissue barriers in other inflammatory diseases [56,57,58]. The validation of MMP9 in the present study further supports ECM degradation and tissue structural damage as important effector mechanisms in the development of gangrenous mastitis.

The WGS results provide additional bacterial context for these host multi-omics findings. The presence of genes encoding LukMF′ and hemolysins is consistent with the pronounced neutrophil-associated and inflammatory responses, whereas adhesins and putative coagulation-interacting factors, including ClfA/ClfB, Coa, and vWbp, may be related to the complement–coagulation and platelet-activation signatures. Similarly, the bacterial protease repertoire is biologically concordant with MMP9-associated extracellular-matrix remodeling and mammary-tissue damage. However, these relationships remain associative because WGS demonstrates genomic potential but does not confirm bacterial gene expression, protein production, or direct causality.

A major limitation of this study is the relatively small sample size of three biological replicates per group, which may have reduced statistical power and generalizability. Moreover, GO/KEGG enrichment indicates associations of differential genes and proteins with particular pathways but does not demonstrate pathway activation or causality. RT-qPCR and Western blotting confirmed expression consistency only; gene function and pathway activity were not experimentally tested. Although the consistency across multiple analyses supports the main biological trends, it cannot replace functional or independent validation. Future studies should therefore include larger cohorts and incorporate functional perturbation and pathway-activity assays.

## 5. Conclusions

Integrated transcriptomic and proteomic analyses showed that *S. aureus*-induced clinical mastitis was characterized primarily by pathogen recognition, acute inflammation, and activation of neutrophil defense responses, whereas gangrenous mastitis involved broader molecular reprogramming. The latter was principally associated with amplification of TNF, IL-17, and NF-κB inflammatory signaling, aberrant neutrophil activation, enhanced complement and coagulation cascades, and ECM remodeling. PPI analysis, GSEA, and validation of key molecules further indicated that IL6, S100A8, THBS1, SERPINE1, and MMP9 may represent important nodes in disease progression. Collectively, gangrenous mastitis is not simply a quantitatively intensified inflammatory response, but a complex infectious tissue-injury process jointly driven by inflammatory amplification, immune-cell-mediated damage, complement–coagulation dysregulation, and disruption of tissue architecture.

## Figures and Tables

**Table 1:** Primer sequences.

Gene Name	Sequences	Product Length
IL6	F: CTTCAGTCCACTCGCTGTCT R: CTGCTTGGGGTGGTGTCATT	108
PTGS2	F: GGCTTCACTTGACCAGAGCA R: CAGCCATTTCCTTCTCTCCTGTA	119
S100A8	F: GTTTTGGGGAGACCTGGTGG R: ACGGCGTGGTAATTCCCTTT	124
THBS1	F: GACTCCGCATTGCCAAAGGA R: GGGTGAGAAAGACACTGGTAGAG	138
SERPINE1	F: CCCTCTACTTCAACGGCCAG R: GTCGGGGGTGGTAAACTCAG	149
MMP9	F: TAGATCACTCCTCCGTGCCA R: GGGCGAGGACCATACAGATG	115
β-actin	F: TCCTCCCTGGAGAAGAGCTAC R: GGTAGTTTCGTGAATGCCGC	135

**Table 2:** Antibodies.

Antibodies	Source	Catalog Number
Anti-IL6	Proteintech	21865-1-AP
Anti-PTGS2	Proteintech	12375-1-AP
Anti-S100A8	Proteintech	15792-1-AP
Anti-THBS1	Proteintech	67241-1-Ig
Anti-SERPINE1	Proteintech	66261-1-Ig
Anti-MMP9	UpingBio	YP-Ab-02639
Anti-β-actin	Proteintech	66009-1-Ig
HRP-conjugated Anti-Mouse	Proteintech	SA00001-1
HRP-conjugated Anti-Rabbit	Proteintech	SA00001-2

**Table 3:** Summary of transcriptomic, proteomic, and integrated differential-expression results.

Comparison	Total DEGs	Total DEPs	Total Concordant Molecules
CLM vs. CON	1085	218	80
GSM vs. CON	3983	678	281
GSM vs. CLM	2303	241	62

**Table 4:** Cross-omics summary of the principal biological findings in each group comparison.

Comparison	Principal Pathways	Representative Molecules
CLM vs. CON	TNF, IL-17, and NF-κB signaling; neutrophil activation	TYROBP, S100A8, S100A9, MMP9, RAC2
GSM vs. CON	Inflammatory signaling; complement and coagulation; HIF-1 signaling; ECM remodeling	IL6, IL1B, S100A8, MMP9, THBS1
GSM vs. CLM	Complement and coagulation; platelet activation; ECM remodeling	IL6, THBS1, SERPINE1, PLAUR, C5

## Data Availability

The original contributions presented in this study are included in the article/Supplementary Materials. Further inquiries can be directed to the corresponding authors.

## Supplementary Materials

The following supporting information can be downloaded at https://www.mdpi.com/article/10.3390/ani16182878/s1. Figure S1. Principal component analysis of mammary gland transcriptomic profiles. Principal component analysis (PCA) was performed on transcriptomic data from the control (CON), clinical mastitis (CLM), and gangrenous mastitis (GSM) groups. Each point represents one biological replicate (*n* = 3 per group), and the colored outlines indicate the distribution of samples within each group. PC1 and PC2 explained 74.92% and 9.08% of the total variance, respectively. Table S1. Whole-genome sequencing and assembly characteristics of the *S. aureus* challenge isolate. Table S2. Major virulence determinants detected in the draft genome.
