# Supplementary material for: Integrated Transcriptomic and Proteomic Analysis of the Pathogenic Mechanisms of Staphylococcus aureus-Induced Gangrenous Mastitis in Dairy Goats
Source: Animals (Basel). 2026 Sep 12;16(18):2878. doi: 10.3390/ani16182878 (PMC13603458; doi:10.3390/ani16182878)
Supplement: Supplementary file 1 [file animals-16-02878-s001.zip › Supplementary Materials/Supplementary Figure S1 Transcriptomic PCA.pdf]

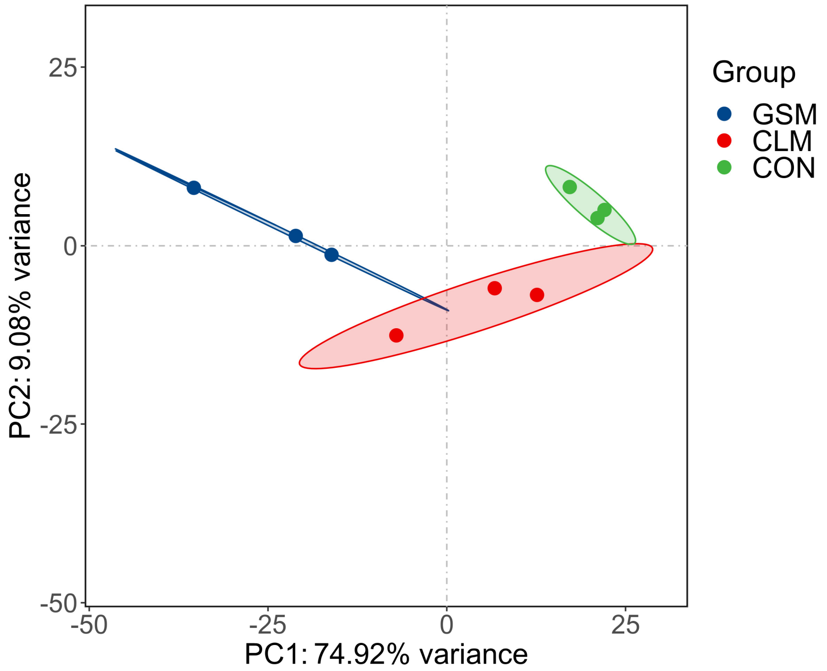

Supplementary Figure. S1. Principal component analysis of mammary gland transcriptomic profiles. Principal component analysis (PCA) was performed on transcriptomic data from the control (CON), clinical mastitis (CLM), and gangrenous mastitis (GSM) groups. Each point represents one biological replicate (*n* = 3 per group), and the colored outlines indicate the distribution of samples within each group. PC1 and PC2 explained 74.92% and 9.08% of the total variance, respectively.
